# Supplementary material for: Residential Radon Exposure and Cigarette Smoking in Association with Lung Cancer: A Matched Case-Control Study in Korea
Source: Int J Environ Res Public Health. 2020 Apr 24;17(8):2946. doi: 10.3390/ijerph17082946 (PMC7215527; doi:10.3390/ijerph17082946)
Supplement: Supplementary file 1 [file ijerph-17-02946-s001.pdf]

Table S1. Associations of residential radon exposure (≥ 74 Bq/m^3) and cigarette smoking with lung cancer.

Variables	Case, n	Control, n	Conditional logistic regression		Conventional logistic regression	
			OR (95% CI)*	*p*-value	OR (95% CI)†	*p*-value
Residential radon						
Low (< 74 Bq/m^3)	364	358	1.00 (reference)		1.00 (reference)	
High (≥ 74 Bq/m^3)	155	161	1.61 (1.16–2.23)	0.005	1.57 (1.13–2.18)	0.007
Cigarette smoking						
Non-smokers	297	338	1.00 (reference)		1.00 (reference)	
Smokers	222	181	2.53 (1.60–3.99)	< 0.001	2.50 (1.59–3.94)	< 0.001
Radon and smoking						
Low-radon non-smokers	208	233	1.00 (reference)		1.00 (reference)	
High-radon non-smokers	89	105	1.55 (1.02–2.34)	0.040	1.55 (1.02–2.35)	0.039
Low-radon smokers	156	125	2.39 (1.45–3.95)	< 0.001	2.41 (1.46–3.96)	< 0.001
High-radon smokers	66	56	4.16 (2.29–7.57)	< 0.001	3.92 (2.16–7.10)	< 0.001

*Adjusted for second-hand smoking, sleeping hours, indoor hours, housing type, floor, presence of house cracks, and green ratio. CI, confidence interval; OR, odds ratio.

†Conventional logistic regression was further adjusted for age and sex.
